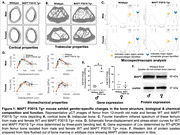# Comorbidity of frontotemporal dementia in the bone evidenced as, gender‐specific alterations in the bone microarchitecture, composition, and biomechanical properties observed in MAPT P301S transgenic mouse model

**DOI:** 10.1002/alz.095656

**Published:** 2025-01-09

**Authors:** Vidyani Suryadevara, Anuradha Kambrath Valiya, Connor J Krehbiel, Swathi Karra, Michael Kluppel, Lisa Miller, Willis S Monte

**Affiliations:** ^1^ Indiana University, Indianapolis, IN USA; ^2^ Stanford University, Stanford, CA USA; ^3^ Rush Univeristy, Chicago, IL USA; ^4^ Brookhaven National Laboratory, Upton, NY USA

## Abstract

**Background:**

Comorbidities are becoming increasingly evident during various Alzheimer’s disease related pathologies. It was found that patients with AD have a higher risk for fractures and falls. Further people who have an incident of falls/fractures have a higher risk for cognitive decline. This study is focused on investigating the alterations in the bone at the structural and functional level in MAPT P301S Tg+ mouse, a preclinical model for frontotemporal dementia.

**Methods:**

The MAPT P301S Tg+ mouse expresses a human 4‐repeat mutant *MAPT P301S* and was developed to model NFTs secondary to tau aggregations. The femur from N = 20 (equal number of male and female) MAPT P301S Tg+ and C57BL/6J mice at 12‐months age were scanned with µCT to characterize the bone microarchitecture. Further three‐point bending test was performed to assess the biomechanical properties and FTIR was used to determine the material properties of the bone. The expression of genes regulating the bone matrix composition was determined by using RTPCR.

**Results:**

The male MAPT P301S Tg+ mice have a significant decrease in bone geometrical parameters (both cortical and trabecular properties) than the females, as compared to their respective wild type. Interestingly the biomechanical properties were altered in both the males and females, wherein there was an increase in the pre‐yield properties, but decrease in the post‐yield properties. This correlated with the alterations in the material properties of the male bone determined by FTIR and the changes in the genes regulating the extracellular matrix of the bone. Tauopathies lead to loss of bone structure and functionality by altering it’s material composition. Further, we found protein expression

**Conclusion:**

This is a first study indicating the presence of Tau in the bone, which impacts the function and this opens up the prospective of Tau impacting other organs, in addition or concurrently with neurodegenerative diseases.